# Association of Obesity Severity With Cardiometabolic and Renal Disease Burden in the United States

**DOI:** 10.1002/oby.70099

**Published:** 2025-11-18

**Authors:** Florina Corpodean, Michael Kachmar, Shengping Yang, Steven B. Heymsfield, Peter T. Katzmarzyk, Philip R. Schauer, Michael W. Cook, Vance L. Albaugh

**Affiliations:** 1 Pennington Biomedical Research Center at Louisiana State University, Baton Rouge, Louisiana, USA; 2 Department of Surgery, Louisiana State University Health Sciences Center, New Orleans, Louisiana, USA

**Keywords:** cardiometabolic disease, high-BMI, obesity

## Abstract

**Objective:**

This study examined the association between obesity severity and cardiometabolic and renal disease, using BMI as a surrogate for obesity severity.

**Methods:**

This is a cross-sectional study using data from the United States Behavioral Risk Factor Surveillance System (BRFSS), 2011–2023. Survey-weighted logistic regression estimated odds ratios (OR) for the diagnosis of diabetes, hypertension, hyperlipidemia, kidney disease, myocardial infarction, stroke, and coronary artery disease among increasing BMI categories.

**Results:**

Higher BMI was associated with increased odds of all conditions. For BMI ≥ 50 kg/m^2^, odds were notably elevated for diabetes (OR 8.32; 95% CI: 7.78–8.91), hypertension (OR 6.07; 95% CI: 5.58–6.61), and kidney disease (OR 3.60; 95% CI: 3.21–4.03). The odds of cardiovascular disease also rose substantially, including myocardial infarction (OR 2.89; 95% CI: 2.56–3.28) and coronary artery disease (OR 3.44; 95% CI: 3.08–3.84). Mean age at diabetes diagnosis decreased with increasing BMI, from 52.2 years in Class I to 45.3 years in Class IV obesity.

**Conclusions:**

Obesity severity is incrementally associated with cardiometabolic and renal disease burden, particularly among adults with BMI ≥ 50 kg/m^2^. These findings highlight the urgent need for early, aggressive interventions targeting individuals with all classes of obesity.

## 1 ∣ Introduction

Obesity prevalence in the United States (U.S.) is rising at an alarming pace [1-4]. While research demonstrates strong associations between obesity (defined as body mass index [BMI] ≥ 30 kg/m^2^) and numerous diseases affecting multiple organ systems [5], the association between obesity severity and disease burden in US adults remains largely unexplored.

Even though recent studies have focused on the clinical significance of obesity and its severity using BMI as a surrogate, unfortunately these analyses also limit “severe obesity” to BMI ≥ 40 kg/m^2^, combine BMI ≥ 30 kg/m^2^ into a single group, and/or focus on a narrow age range [6-9]. These practices are concerning, since the fastest growing categories of severe obesity in the US are BMI ≥ 50 kg/m^2^ and higher BMI subcategories [9-12]. Better appreciation of these higher BMI populations is critical, especially as these patients have increased complexity and present significant clinical challenges [13]. Thus, the objective of this study was to test the hypothesis that obesity severity is associated with higher cardiometabolic and renal disease prevalence.

## 2 ∣ Methods

A cross-sectional analysis was conducted using data from the U.S. Behavioral Risk Factor Surveillance System (BRFSS) collected between 2011 and 2023. BRFSS is a nationally representative, telephone-based health survey administered by the Centers for Disease Control and Prevention (CDC), which collects self-reported information on health-related risk behaviors, chronic conditions, and preventive health practices among U.S. adults. After excluding individuals with missing BMI, the final sample included over 5 million adults aged 18 years or older. To assess the impact of missing disease-specific data, a sensitivity analysis including only complete case data (*n* = 2,257,974) was performed (online Supporting Information), which showed nearly identical results as the primary analysis (*n* = 5,327,214).

BMI was used as a surrogate for obesity severity and categorized as follows: 30.0–34.9 kg/m^2^ (Class I), 35.0–39.9 kg/m^2^ (Class II), 40.0–49.9 kg/m^2^ (Class III), and ≥ 50.0 kg/m^2^ (Class IV and greater). Individuals with a non-obesity BMI (18.5–29.9 kg/m^2^) served as the reference group. BMI ≥ 50.0 kg/m^2^ was used as the highest BMI group empirically, given the incompatible sample size of individuals at higher BMI groupings for the BRFSS complex survey design.

Survey-weighted logistic regression estimated the odds of cardiometabolic and renal conditions (Table 1) across BMI categories. All models were adjusted for age (continuous), sex (female/male), and race (White/Caucasian, Black/African American, other). Models examining the extent of cardiovascular disease conditions (history of myocardial infarction, stroke, coronary artery disease) were also adjusted for hypertension and hyperlipidemia, known risk factors for coronary artery disease. Similarly, kidney disease models were also adjusted for hypertension and diabetes. Insulin use among participants with diabetes was evaluated using logistic regression, and linear regression was used to examine the association between BMI category and age at diabetes diagnosis. Age at diagnosis was only available for diabetes.

All analyses incorporated BRFSS survey weights, primary sampling units (PSUs), and stratification variables to ensure nationally representative estimates. Statistical significance was defined as *p* < 0.0001.

## 3 ∣ Results

**FIGURE 1 ∣:**
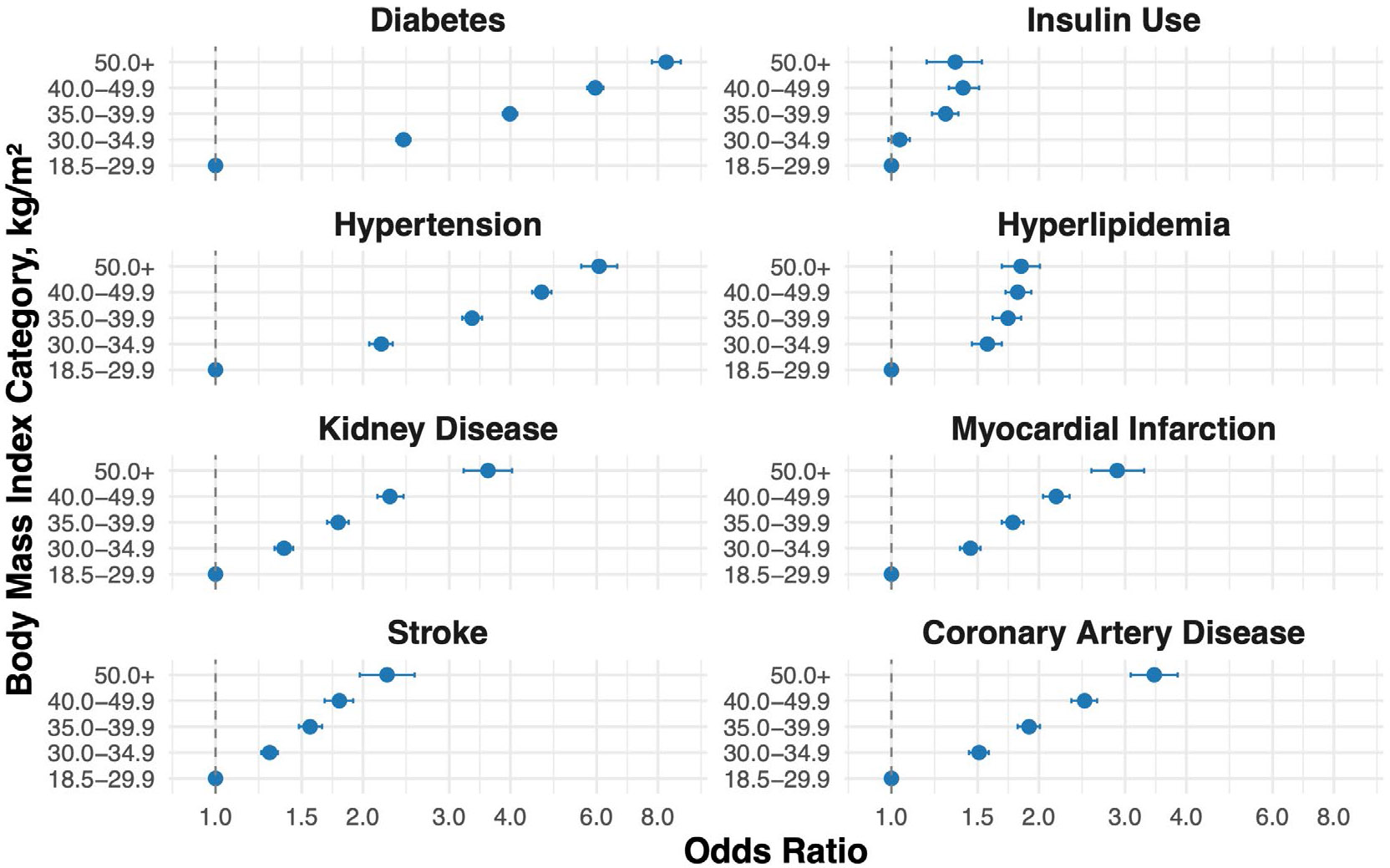
Odds ratios for the association of BMI categories with cardiometabolic and renal conditions: Survey-weighted logistic regression models were used to calculate odds ratios for eight chronic conditions across BMI categories (30.0–34.9, 35.0–39.9, 40.0–49.9, and ≥ 50 kg/m^2^), with individuals in the BMI 18.5–29.9 kg/m^2^ range serving as the reference. Blue circles represent age, sex, and race adjusted models. Error bars represent 95% CI. All models are adjusted for age, sex, and race.

**FIGURE 2 ∣:**
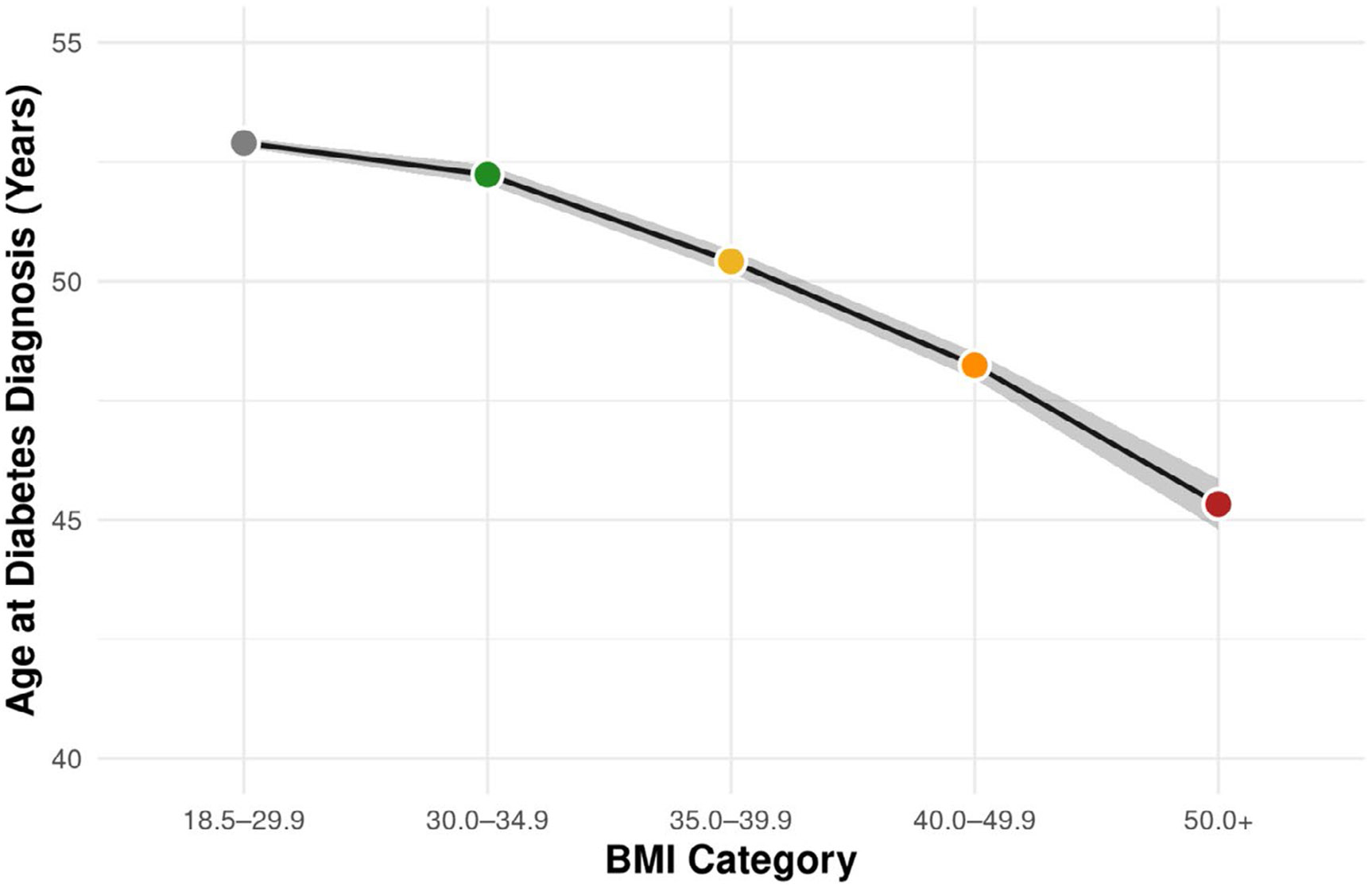
Association of obesity severity with mean age at diabetes diagnosis. Survey-weighted linear regression was used to estimate the mean age at diabetes diagnosis across BMI categories (30.0–34.9, 35.0–39.9, 40.0–49.9, and ≥ 50 kg/m^2^). Points represent weighted means with 95% CI shown as shaded areas. All mean ages at diabetes diagnosis differed significantly from the reference (BMI 18.5–29.9).

Compared to individuals without obesity (BMI 18.5–29.9 kg/m^2^), those in higher BMI categories had progressively greater odds of cardiometabolic and renal disease (Table 2, Figure 1). Odds of having diabetes markedly increased across BMI categories, from 2.42 (95% CI: 2.34–2.50) for BMI 30.0–34.9 to 8.32 (95% CI: 7.78–8.91) for BMI ≥ 50. Insulin use was also more likely with higher BMI, reaching an odds ratio of 1.35 (95% CI: 1.18–1.53) in the highest BMI group. In addition to differences in disease burden, obesity severity was associated with a younger mean age at diabetes diagnosis (Figure 2). Individuals in the BMI 30.0–34.9 group were diagnosed with diabetes at a mean age of 52.2 years compared to 45.3 years among those with BMI ≥ 50.

The odds of hypertension rose from 2.18 (95% CI: 2.06–2.30) in the BMI 30.0–34.9 group to 6.07 (95% CI: 5.58–6.61) among those with BMI ≥ 50. For hyperlipidemia, odds increased across all BMI categories compared to reference, without a stepwise increase across BMI categories. The odds ranged from 1.57 (95% CI: 1.46–1.68) for BMI 30.0–34.9 to 1.81 (95% CI: 1.71–1.93) for BMI 40.0–49.9 and remained elevated in the BMI ≥ 50 group (OR: 1.84; 95% CI: 1.68–2.01). Kidney disease showed a strong association with obesity severity, with odds increasing from 1.38 (95% CI: 1.32–1.44) to 3.60 (95% CI: 3.21–4.03); significant but slightly attenuated associations remained after adjustment for hypertension and diabetes (OR 2.01; 95% CI: 1.73–2.34 for BMI ≥ 50).

Adverse cardiovascular conditions followed a similar pattern overall, with individuals with BMI ≥ 50 having over twice the odds of myocardial infarction (OR: 2.89; 95% CI: 2.56–3.28), stroke (OR: 2.24; 95% CI: 1.97–2.55), and coronary artery disease (OR: 3.44; 95% CI: 3.08–3.84) compared to reference. After adjustment for comorbid hypertension and hyperlipidemia, these associations persisted though were slightly attenuated (e.g., coronary artery disease OR: 2.27; 95% CI: 1.97–2.62).

Race and sex showed significant effects in all models. Independent of BMI, Black adults had higher odds of diabetes (OR: 1.79; 95% CI: 1.74–1.84), insulin use (OR: 1.23; 95% CI: 1.16–1.31), kidney disease (OR: 1.32; 95% CI: 1.26–1.39), hypertension (OR: 1.98; 95% CI: 1.90–2.05), stroke (OR: 1.80; 95% CI: 1.73–1.88), and myocardial infarction (OR: 1.17; 95% CI: 1.12–1.23) compared to White adults. Relative to males in the sample, females collectively had relatively lower odds of myocardial infarction (OR: 0.48; 95% CI: 0.46–0.50), hypertension (OR: 0.71; 95% CI: 0.65–0.77), coronary disease (OR: 0.58; 95% CI: 0.56–0.60), and insulin use (OR: 0.93; 95% CI: 0.89–0.97), but higher odds of kidney disease (OR: 1.10; 95% CI: 1.06–1.15). To better understand sex-specific effects of increasing BMI on disease burden, separate models were analyzed for males and females that revealed similar increases in odds of cardiometabolic and renal diseases as seen in the complete sample (Table S3).

## 4 ∣ Discussion

Leveraging nationally representative data, this analysis highlights the strong and graded association between obesity severity and cardiometabolic and renal disease burden in U.S. adults—offering novel insights into an often-overlooked population with significant implications for risk stratification and disease management [9, 10, 12]. While stepwise increases in the odds of most comorbidities were observed across increasing BMI categories, increased prevalence was even detectable for Class I obesity, a concerning finding. For example, the odds of having diabetes were over two-fold higher in the Class I obesity group compared to individuals without obesity. These findings highlight the fact that the clinical impact of obesity emerges early in the disease, emphasizing the need for early intervention and preventive measures.

As BMI is a surrogate for excess adiposity, this study provides strong evidence that the degree of adiposity may drive marked differences in cardiometabolic risk. While the exact mechanisms underlying the relationship between BMI and cardiometabolic disease burden remain unclear, numerous studies suggest that increased adiposity is associated with lipotoxicity, insulin resistance, vascular dysfunction, and higher circulating adipokines and other inflammatory mediators [14]—all of which contribute to cardiometabolic risk. Future work must focus on understanding the pathophysiologic effects of high degrees of adiposity, especially with respect to higher BMI individuals [12].

Key strengths of this study include the large, nationally representative sample and stratification of obesity severity by BMI, enabling generalizable estimates. Notably, the inclusion of individuals with BMI ≥ 50 kg/ms offers valuable insights into a high-risk group often excluded from research, despite their increasing prevalence in the U.S. population. Importantly, these individuals also experience heightened weight stigma, implicit provider bias, and structural barriers to care—all contributing factors to poor health outcomes. Such biases may discourage healthcare utilization, further compounding disease risk and health disparities. As a result, individuals with severe obesity may represent not only a metabolically high-risk population, but one that is systematically underserved.

This study also underscores racial disparities present in obesity-related health outcomes. Black adults had significantly higher odds of diabetes, hypertension, kidney disease, and stroke, even after adjusting for BMI. These findings suggest that obesity interacts with broader social and structural factors that disproportionately impact Black individuals and mirror findings from other recent studies [15]. Targeted interventions are needed to expand access to care and address systemic inequities in healthcare delivery.

Limitations of this study include the use of self-reported data with the potential for reporting and recall bias, particularly for BMI estimation. However, prior research demonstrates a consistent tendency for BMI underreporting, especially with increasing BMI [9, 10, 16]. Despite the marked increases in odds of cardiometabolic disease observed, health literacy also decreases with increasing cardiometabolic disease burden, suggesting these findings could be underestimates [17, 18]. Additionally, cross-sectional design precludes causal inference and limits the ability to evaluate the temporal relationship between BMI and disease onset. Furthermore, while the National Health and Nutrition Examination Survey (NHANES) demonstrates the fastest growing BMI category in the U.S. to be BMI > 60 kg/m^2^ [12], the sample size of this group in the BRFSS was too small to examine this group individually. This is likely because NHANES includes direct measures of height and weight, whereas BRFSS relies on self-reported data that could be even less accurate for individuals at very high BMI. Finally, while alternatives to the use of BMI are increasingly discussed with respect to obesity diagnosis [19], BMI remains a highly accurate surrogate for excess adiposity associated with clinical and preclinical obesity [20].

## 5 ∣ Conclusion

Further research must support the development of effective clinical and public health strategies for treating obesity as a means of preventing cardiometabolic and renal diseases. Individuals in the highest BMI categories especially benefit from early identification and comprehensive, intensive obesity management to reduce long-term complications and improve health outcomes.

## Supplementary Material

supinfo

Additional supporting information can be found online in the Supporting Information section. **Supporting Information**. A complete-case sensitivity analysis was conducted to examine the potential effect of missing data on model estimates. Results were consistent with the main analysis (Table S1). Using the complete data set, we conducted a comorbidity-adjusted model (Table S2). Additional sex-specific analyses are summarized in Table S3.

## Figures and Tables

**TABLE 1 ∣:** Definitions of BRFSS health conditions.

Condition	Definition
Diabetes	Respondent has ever been told by a doctor, nurse, or other health professional that they have diabetes. If female and the response is “Yes,” a follow-up question determines if it was only during pregnancy.
Insulin use	Among respondents who have been told they have diabetes, this variable indicates whether they are currently taking insulin.
Hypertension	Respondent has ever been told by a doctor, nurse, or other health professional that they have high blood pressure.
Kidney disease	Respondent has ever been told by a doctor, nurse, or other health professional that they have kidney disease. This does not include kidney stones, bladder infections, or incontinence.
Hyperlipidemia	Respondent has ever been told by a doctor, nurse, or other health professional that their blood cholesterol is high.
Myocardial infarction	Respondent has ever been told by a doctor, nurse, or other health professional that they had a heart attack, also called a myocardial infarction.
Stroke	Respondent has ever been told by a doctor, nurse, or other health professional that they had a stroke.
Coronary artery disease	Respondent has ever been told by a doctor, nurse, or other health professional that they had angina or coronary heart disease.

**TABLE 2 ∣:** Unadjusted and adjusted odds ratios for cardiometabolic and renal disease by BMI category.

Unadjusted odds ratios				
Condition	BMI 30.0–34.9	BMI 35.0–39.9	BMI 40.0–49.9	BMI 50.0+
Diabetes	2.37 (2.34–2.40)***	3.53 (3.48–3.59)***	4.65 (4.56–4.74)***	5.63 (5.41–5.86)***
Insulin use	1.05 (1.01–1.09)***	1.28 (1.22–1.34)***	1.44 (1.37–1.51)***	1.53 (1.39–1.68)***
Hypertension	2.13 (2.03–2.25)***	2.86 (2.71–3.01)***	3.45 (3.26–3.64)***	3.81 (3.54–4.10)***
Kidney disease	1.48 (1.45–1.51)***	1.78 (1.75–1.82)***	2.13 (2.08–2.17)***	2.92 (2.76–3.09)***
Hyperlipidemia	1.56 (1.51–1.60)***	1.62 (1.57–1.67)***	1.55 (1.50–1.61)***	1.38 (1.30–1.46)***
Myocardial infarction	1.46 (1.43–1.49)***	1.58 (1.54–1.61)***	1.57 (1.53–1.61)***	1.71 (1.59–1.85)***
Stroke	1.31 (1.28–1.34)***	1.47 (1.43–1.52)***	1.58 (1.53–1.64)***	1.76 (1.63–1.91)***
Coronary artery disease	1.51 (1.49–1.54)***	1.67 (1.64–1.70)***	1.77 (1.73–1.82)***	1.98 (1.86–2.11)***
Adjusted odds ratios				
Condition	BMI 30.0–34.9	BMI 35.0–39.9	BMI 40–49.9	BMI 50+
Diabetes	2.42 (2.34–2.50)***	3.99 (3.86–4.13)***	5.96 (5.74–6.19)***	8.32 (7.78–8.91)***
Insulin use	1.04 (0.99–1.09)	1.29 (1.21–1.37)***	1.40 (1.31–1.51)***	1.35 (1.18–1.53)***
Hypertension	2.18 (2.06–2.30)***	3.34 (3.19–3.50)***	4.63 (4.43–4.85)***	6.07 (5.58–6.61)***
Kidney disease	1.38 (1.32–1.44)***	1.78 (1.69–1.87)***	2.27 (2.14–2.42)***	3.60 (3.21–4.03)***
Hyperlipidemia	1.57 (1.46–1.68)***	1.73 (1.61–1.84)***	1.81 (1.71–1.93)***	1.84 (1.68–2.01)***
Myocardial infarction	1.45 (1.38–1.52)***	1.77 (1.68–1.86)***	2.17 (2.04–2.31)***	2.89 (2.56–3.28)***
Stroke	1.29 (1.24–1.34)***	1.56 (1.48–1.65)***	1.79 (1.67–1.91)***	2.24 (1.97–2.55)***
Coronary artery disease	1.51 (1.44–1.58)***	1.91 (1.81–2.01)***	2.48 (2.33–2.63)***	3.44 (3.08–3.84)***

*Note:* Data from the Behavioral Risk Factor Surveillance System (BRFSS) with a BMI reference group of 18.5–29.9 kg/m^2^. Data are the odds ratios and 95% CI. The p value corresponds to the impact of the BMI category on the likelihood of having a cardiometabolic or renal diagnosis compared to the reference. Adjusted models control for age, sex, and race. Statistical significance, ***p < 0.0001.

## Data Availability

The data that support the findings of this study are available in the Behavioral Risk Factor Surveillance System. These data were derived from the following resources available in the public domain: CDC Website, https://www.cdc.gov/brfss/index.html.

## Study Importance

- What is already known?
  - Obesity increases the risk of diabetes, hypertension, and heart disease, but most studies group all obesity together or stop at Class III (BMI ≥ 40 kg/m^2^), failing to capture the unique risks faced by those with more severe obesity.
- What does this study add?
  - A clear, stepwise increase in cardiometabolic and kidney disease risk is associated with higher BMI, starting as early as Class I.
  - Adults with BMI ≥ 50 face dramatically higher odds and are diagnosed with diabetes at younger ages.
- How might these results change the direction of research or the focus of clinical practice?
  - These findings support earlier and more tailored intervention across all obesity classes and highlight the urgent need to address the unmet medical and structural needs of individuals with severe obesity.
  - A clinical and research focus on pathophysiology and treatment for higher BMI (> 50 kg/m^2^) individuals is needed.

## References

[1] Ward ZJ, Bleich SN, Cradock AL, , “Projected U.S. State-Level Prevalence of Adult Obesity and Severe Obesity,” New England Journal of Medicine 381 (2019): 2440–2450. 31851800 10.1056/NEJMsa1909301

[2] Zhang X, Liu J, Ni Y, , “Global Prevalence of Overweight and Obesity in Children and Adolescents,” JAMA Pediatrics 178 (2024): 800–813. 38856986 10.1001/jamapediatrics.2024.1576 PMC11165417

[3] Emmerich S, Fryar C, Stierman B, and Ogden C, “Obesity and Severe Obesity Prevalence in Adults: United States, August 2021–August 2023,” NCHS Data Brief (2024), 10.15620/cdc/159281. PMC11744423 39808758

[4] Münte E, Zhang X, Khurana A, and Hartmann P, “Prevalence of Extremely Severe Obesity and Metabolic Dysfunction Among US Children and Adolescents,” JAMA Network Open 8 (2025): e2521170. 40668581 10.1001/jamanetworkopen.2025.21170 PMC12268495

[5] Kivimäki M, Strandberg T, Pentti J, , “Body-Mass Index and Risk of Obesity-Related Complex Multimorbidity: An Observational Multicohort Study,” Lancet Diabetes and Endocrinology 10 (2022): 253–263. 35248171 10.1016/S2213-8587(22)00033-X PMC8938400

[6] Yao Z, Tchang BG, Albert M, Blumenthal RS, Nasir K, and Blaha MJ, “Associations Between Class I, II, or III Obesity and Health Outcomes,” NEJM Evidence 4 (2025): EVIDoa2400229. 40130972 10.1056/EVIDoa2400229

[7] Rassy N, Straaten AV, Carette C, Hamer M, Rives-Lange C, and Czernichow S, “Association of Healthy Lifestyle Factors and Obesity-Related Diseases in Adults in the UK,” JAMA Network Open 6 (2023): e2314741. 37234008 10.1001/jamanetworkopen.2023.14741 PMC10220514

[8] Gearon E and Backholer K, “Demographic Prevalence of Class III Obesity,” JAMA Internal Medicine 175 (2015): 2000–2001. 10.1001/jamainternmed.2015.6533 26641361

[9] Hattori A and Sturm R, “The Obesity Epidemic and Changes in SelfReport Biases in BMI,” Obesity 21 (2013): 856–860. 23712990 10.1002/oby.20313 PMC5800501

[10] Sa C, Pe M, Ml A, and Sj A, “Cardiometabolic Risks and Severity of Obesity in Children and Young Adults,” New England Journal of Medicine 373 (2015): 1307–1317. 26422721 10.1056/NEJMoa1502821

[11] Sturm R and Hattori A, “Morbid Obesity Rates Continue to Rise Rapidly in the United States,” International Journal of Obesity 37 (2013): 889–891. 22986681 10.1038/ijo.2012.159 PMC3527647

[12] Kachmar M, Albaugh VL, Yang S, , “Disproportionate Increase in BMI of ≥ 60 Kg/m^2^ in the USA,” Lancet Diabetes and Endocrinology 13 (2025): 463–465. 40288378 10.1016/S2213-8587(25)00069-5

[13] Corpodean F, Kachmar M, Yang S, Cook MW, Schauer PR, and Albaugh VL, “Obesity Severity and Cancer Screening in US Adults,” JAMA Network Open 8 (2025): e2532402. 40960829 10.1001/jamanetworkopen.2025.32402 PMC12444554

[14] Valenzuela PL, Carrera-Bastos P, Castillo-García A, Lieberman DE, Santos-Lozano A, and Lucia A, “Obesity and the Risk of Cardiometabolic Diseases,” Nature Reviews. Cardiology 20 (2023): 475–494. 36927772 10.1038/s41569-023-00847-5

[15] Lofton H, Ard JD, Hunt RR, and Knight MG, “Obesity Among African American People in the United States: A Review,” Obesity 31 (2023): 306–315. 36695059 10.1002/oby.23640 PMC10107750

[16] Flegal KM, Kruszon-Moran D, Carroll MD, Fryar CD, and Ogden CL, “Trends in Obesity Among Adults in the United States, 2005 to 2014,” JAMA 315 (2016): 2284–2291. 10.1001/jama.2016.6458 PMC11197437 27272580

[17] Tajdar D, Schäfer I, Lühmann D, , “The Link Between Health Literacy and Three Conditions of Metabolic Syndrome: Obesity, Diabetes and Hypertension,” Diabetes, Metabolic Syndrome and Obesity: Targets and Therapy 15 (2022): 1639–1650. 35651900 10.2147/DMSO.S363823 PMC9150919

[18] Michou M, Panagiotakos DB, and Costarelli V, “Low Health Literacy and Excess Body Weight: A Systematic Review,” Central European Journal of Public Health 26 (2018): 234–241. 30419628 10.21101/cejph.a5172

[19] Rubino F, Batterham RL, Koch M, , “Lancet Diabetes & Endocrinology Commission on the Definition and Diagnosis of Clinical Obesity,” Lancet Diabetes and Endocrinology 11 (2023): 226–228. 36878238 10.1016/S2213-8587(23)00058-X

[20] Aryee EK, Zhang S, Selvin E, and Fang M, “Prevalence of Obesity With and Without Confirmation of Excess Adiposity Among US Adults,” JAMA 333 (2025): 1726–1728. 40244602 10.1001/jama.2025.2704 PMC12006908

