# Supplementary material for: Association of Obesity Severity With Cardiometabolic and Renal Disease Burden in the United States
Source: Obesity (Silver Spring). 2025 Nov 18;34(2):323–8. doi: 10.1002/oby.70099 (PMC12834209; doi:10.1002/oby.70099)
Supplement: Supplementary file 1 — Supporting Information. A complete‐case sensitivity analysis was conducted to examine the potential effect of missing data on model estimates. Results were consistent with the main analysis (Table S1). Using the complete data set, we conducted a comorbidity‐adjusted model (Table S2). Additional sex‐specific analyses are summarized in Table S3. [file OBY-34-323-s001.docx]

**SUPPLEMENTAL MATERIALS**

**for**

**Association of Obesity Severity with Cardiometabolic and Renal Disease Burden in the United States**

Florina Corpodean^1,2^, Michael Kachmar^1,2^, Shengping Yang^1^, Steven B. Heymsfield^1^, Peter T. Katzmarzyk^1^, Philip R. Schauer^1,2^, Michael W. Cook^2^, Vance L. Albaugh^1,2^

^1^Pennington Biomedical Research Center at Louisiana State University, Baton Rouge, LA, USA

^2^Department of Surgery, Louisiana State University Health Sciences Center, New Orleans, LA, USA

**Corresponding Author:**

Vance L. Albaugh, MD, PhD

Pennington Biomedical Research Center

6400 Perkins Rd

Baton Rouge, LA, USA

vance.albaugh@pbrc.edu

P: (225) 763-2500

F: (225) 763-2525

**SUPPLEMENTAL MATERIALS**

A complete-case sensitivity analysis was conducted to examine the potential effect of missing data on model estimates. Results were consistent with the main analysis (**Supplemental Table S1).** Using the complete data set, we conducted a comorbidity-adjusted model (**Supplemental** **Table S2).** Additional sex-specific analyses are summarized in **Supplemental Table S3.**

**Supplemental Table S1. Complete Case Adjusted Odds Ratios of Cardiometabolic and Renal Disease**

| **Condition** | **BMI 30.0-34.9** | **BMI 35.0-39.9** | **BMI 40.0-49.9** | **BMI 50.0+** |
| --- | --- | --- | --- | --- |
| Diabetes | 2.67 (2.56-2.79)*** | 4.58 (4.37-4.80)*** | 6.66 (6.33-7.01)*** | 9.38 (8.63-10.2)*** |
| Insulin Use | 1.04 (0.99-1.09) | 1.24 (1.17-1.31)*** | 1.36 (1.27-1.46)*** | 1.46 (1.28-1.66)*** |
| Hypertension | 2.15 (2.02-2.30)*** | 3.19 (3.00-3.39)*** | 4.04 (3.80-4.30)*** | 5.29 (4.87-5.74)*** |
| Kidney Disease | 1.39 (1.32-1.46)*** | 1.80 (1.70-1.91)*** | 2.37 (2.21-2.55)*** | 3.44 (3.02-3.91)*** |
| Hyperlipidemia | 1.55 (1.47-1.63)*** | 1.68 (1.60-1.77)*** | 1.63 (1.55-1.72)*** | 1.59 (1.47-1.72)*** |
| Myocardial Infarction | 1.45 (1.39-1.52)*** | 1.69 (1.59-1.79)*** | 1.82 (1.70-1.96)*** | 2.05 (1.78-2.35)*** |
| Stroke | 1.30 (1.24-1.37)*** | 1.54 (1.45-1.63)*** | 1.83 (1.69-1.98)*** | 2.30 (1.97-2.69)*** |
| Coronary Artery Disease | 1.52 (1.46-1.60)*** | 1.85 (1.75-1.96)*** | 2.08 (1.94-2.23)*** | 2.43 (2.14-2.78)*** |
| Note: Data from the Behavioral Risk Factor Surveillance System (BRFSS) with a BMI reference group of 18.5-29.9 kg/m2. Data are the odds ratios and 95% confidence intervals. The *p*-value corresponds to the impact of BMI category on the likelihood of having a cardiometabolic or renal diagnosis compared to the reference BMI group. Statistical significance ****p*<0.0001. | | | | |

**Supplemental Table S2: Comorbidity-Adjusted^‡^ Odds Ratios of Cardiometabolic and Renal Disease**

| **Condition** | **BMI 30-34.9** | **BMI 35-39.9** | **BMI 40-49.9** | **BMI 50+** |
| --- | --- | --- | --- | --- |
| Kidney Disease | 1.06 (0.99-1.12) | 1.18 (1.10-1.27)*** | 1.35 (1.24-1.47)*** | 2.01 (1.73-2.34)*** |
| Myocardial Infarction | 1.21 (1.13-1.29)*** | 1.35 (1.25-1.46)*** | 1.57 (1.43-1.72)*** | 2.03 (1.73-2.38)*** |
| Stroke | 1.13 (1.06-1.20)*** | 1.19 (1.10-1.29)*** | 1.37 (1.24-1.51)*** | 1.72 (1.45-2.05)*** |
| Coronary Artery Disease | 1.24 (1.16-1.32)*** | 1.47 (1.37-1.59)*** | 1.75 (1.61-1.90)*** | 2.27 (1.97-2.62)*** |
| Note: Data from the Behavioral Risk Factor Surveillance System (BRFSS) with a BMI reference of 18.5-29.9 kg/m2. Data are the odds ratios and 95% confidence intervals. The *p*-value corresponds to the impact of BMI category on the likelihood of having a cardiometabolic or renal diagnosis compared to the reference BMI group. **^‡^**Models were adjusted for age, sex, race as well as associated comorbidities (Hypertension and Hyperlipidemia for Stroke, Myocardial Infarction and Coronary Artery Disease, Hypertension and Diabetes for Kidney Disease). Statistical significance ****p*<0.0001. | | | | |

**Supplemental Table S3: Adjusted Odds Ratios of Cardiometabolic and Renal Disease by Sex**

| **Outcome** | **BMI** | **OR Male** | **OR Female** |
| --- | --- | --- | --- |
| Diabetes | 30.0-34.9 | 2.37 (2.22-2.54) | 2.53 (2.46-2.59) |
| Diabetes | 35.0-39.9 | 4.17 (3.88-4.48) | 4.01 (3.88-4.13) |
| Diabetes | 40.0-49.9 | 7.08 (6.56-7.65) | 5.62 (5.41-5.84) |
| Diabetes | 50+ | 9.42 (8.22-10.8) | 8.13 (7.53-8.77) |
| Insulin Use | 30.0-34.9 | 1.01 (0.94-1.09) | 1.07 (1.00-1.15) |
| Insulin Use | 35.0-39.9 | 1.29 (1.18-1.41) | 1.31 (1.20-1.42) |
| Insulin Use | 40.0-49.9 | 1.52 (1.35-1.70) | 1.36 (1.24-1.49) |
| Insulin Use | 50+ | 1.23 (0.98-1.56) | 1.44 (1.23-1.68) |
| Hypertension | 30.0-34.9 | 2.05 (1.89-2.22) | 2.43 (2.36-2.49) |
| Hypertension | 35.0-39.9 | 3.29 (3.05-3.55) | 3.66 (3.53-3.80) |
| Hypertension | 40.0-49.9 | 4.63 (4.26-5.03) | 5.24 (4.98-5.52) |
| Hypertension | 50+ | 5.47 (4.68-6.40) | 7.41 (6.66-8.24) |
| Hyperlipidemia | 30.0-34.9 | 1.53 (1.36-1.72) | 1.57 (1.53-1.61) |
| Hyperlipidemia | 35.0-39.9 | 1.68 (1.49-1.89) | 1.75 (1.69-1.82) |
| Hyperlipidemia | 40.0-49.9 | 1.67 (1.47-1.88) | 1.92 (1.82-2.01) |
| Hyperlipidemia | 50+ | 1.60 (1.35-1.89) | 1.98 (1.78-2.20) |
| Kidney Disease | 30.0-34.9 | 1.35 (1.26-1.45) | 1.43 (1.36-1.50) |
| Kidney Disease | 35.0-39.9 | 1.81 (1.65-1.97) | 1.81 (1.70-1.92) |
| Kidney Disease | 40.0-49.9 | 2.36 (2.11-2.65) | 2.27 (2.12-2.44) |
| Kidney Disease | 50+ | 3.61 (2.94-4.43) | 3.66 (3.19-4.19) |
| Myocardial Infarction | 30.0-34.9 | 1.43 (1.33-1.54) | 1.47 (1.41-1.53) |
| Myocardial Infarction | 35.0-39.9 | 1.75 (1.62-1.90) | 1.79 (1.69-1.90) |
| Myocardial Infarction | 40.0-49.9 | 2.23 (2.02-2.46) | 2.13 (1.99-2.29) |
| Myocardial Infarction | 50+ | 2.81 (2.30-3.44) | 2.97 (2.54-3.47) |
| Stroke | 30.0-34.9 | 1.24 (1.15-1.33) | 1.38 (1.33-1.44) |
| Stroke | 35.0-39.9 | 1.44 (1.31-1.58) | 1.74 (1.63-1.85) |
| Stroke | 40.0-49.9 | 1.82 (1.60-2.05) | 1.92 (1.77-2.07) |
| Stroke | 50+ | 2.32 (1.86-2.88) | 2.40 (2.04-2.81) |
| Coronary Artery Disease | 30.0-34.9 | 1.46 (1.36-1.58) | 1.57 (1.51-1.63) |
| Coronary Artery Disease | 35.0-39.9 | 1.84 (1.68-2.01) | 1.99 (1.89-2.09) |
| Coronary Artery Disease | 40.0-49.9 | 2.40 (2.16-2.66) | 2.53 (2.37-2.70) |
| Coronary Artery Disease | 50+ | 3.23 (2.69-3.88) | 3.53 (3.08-4.03) |
| Note: Data from the Behavioral Risk Factor Surveillance System (BRFSS) with a BMI reference of 18.5-29.9 kg/m^2^. Data are the odds ratios and 95% confidence intervals. Sex-stratified models were fit separately in men and women, adjusting for age and race. Statistical significance was defined as p<0.05, with strong significance indicated by p<0.001 and p<0.0001. Abbreviations: OR, odds ratio; BMI, body mass index. | | | |
